# Opposite Influence of Light and Blindness on Pituitary–Gonadal Function

**DOI:** 10.3389/fendo.2013.00205

**Published:** 2014-01-13

**Authors:** Antonio Bellastella, Annamaria De Bellis, Giuseppe Bellastella, Katherine Esposito

**Affiliations:** ^1^Department of Cardiothoracic and Respiratory Sciences, Second University of Naples, Naples, Italy; ^2^Department of Medical, Surgical, Neurological, Metabolic and Geriatric Sciences, Second University of Naples, Naples, Italy; ^3^Department of Clinical and Experimental Medicine, Second University of Naples, Naples, Italy

**Keywords:** light, blindness, clock genes, melatonin, pituitary–gonadal function

## Abstract

Some environmental factors may influence the pituitary–gonadal function. Among these, light plays an important role in animals and in humans. The effect of light on the endocrine system is mediated by the pineal gland, through the modulation of melatonin secretion. In fact, melatonin secretion is stimulated by darkness and suppressed by light, thus its circadian rhythm peaks at night. Light plays a favorable action on the hypothalamic-pituitary axis likely inhibiting melatonin secretion, while the exogenous melatonin administration does not seem to impair the hormonal secretions of this axis. The basal and rhythmic pituitary–gonadal hormone secretions are regulated by a central clock gene and some independent clock genes in the peripheral tissues. Light is able to induce the expression of some of these genes, thus playing an important role in regulating the hormonal secretions of pituitary–gonadal axis and the sexual and reproductive function in animals and humans. The lack of light stimulus in blind subjects induces increase in plasma melatonin concentrations with a free-running rhythm of secretion, which impairs the hormonal secretions of pituitary–gonadal axis, causing disorders of reproductive processes in both sexes.

## Introduction

Several endogenous and exogenous factors may influence endocrine secretions (1), including those of pituitary–gonadal axis (2). Among the exogenous environmental factors, light seems to play a pivotal role both in animals and in humans, especially as synchronizing agent of hormonal rhythmicity (3–5). Several structures are involved in the mechanism of transmission of light stimulus to the circadian timing system: a retinal component with photoreceptor and ganglion cells, a retino-hypothalamic tract (RHT) originating from these and projected to the suprachiasmatic nucleus (SCN), the circadian pacemaker, i.e., the SCN, efferent projections of SCN to a series of hypothalamic and thalamic nuclei (6). The major projections are to areas that themselves receive retinal input and project reciprocally to the SCN. Of particular importance are the projections of the SCN that reach the supraventricular zone and then the hypothalamus because they provide, among other functions, the neuroendocrine regulation and the pineal melatonin secretion, which plays an intermediate role between the environment and the endocrine system. Studies on the effects of light on the endocrine secretions in animals are usually performed by exposing them to different photoperiods or rendering them blind. In humans, blindness may be considered, despite unlucky, an experimental condition to study the effects of light on the hormonal secretions, but in this regard data are scarce and sometimes controversial. However, since light is one of the most important environmental factors, paying attention to its influence on the endocrine system may avoid misleading interpretation of individual hormonal data and may help prevent alterations in hormonal pattern and rhythmicity caused by variations of this environmental entraining-agent.

## Molecular Aspects

The recent identification of several clock genes in a number of organism, including mammals (7–14), seems to assign a pivotal role to the hypothalamus as pacemaker of pituitary–gonadal secretions. However, the findings of independent clocks in peripheral tissues (1, 9, 12–15) suggest a possible gonadal independent role in regulating the rhythmicity of gonadal steroids. In fact, recent findings support the assumption that some clock genes can influence fertility and testosterone (T) seasonality both in animals (16) and in humans (17). In particular, *Brain and muscle Arnt-like protein 1 (BMAL1)* and *Neuronal PAS domain protein 2 (NPAS2)* gene variants have been shown to influence fertility and seasonality in humans (17). Anyway, since light plays an important synchronizing role on the circadian rhythmicity, the alteration of photoperiod, or the lack of light stimulus, as occurring in blindness, may impair this rhythmicity (18). Consequently, the desynchronizing effect of altered light signal may influence circadian peripheral clocks in female and male reproductive tissues causing impairment of fertility (19) with disorders in estrus cycles, ovulation, sperm generation, implantation, and the progression of pregnancy (14).

In fact, light may act at molecular level inducing the expression of some immediate early genes in the SCN involved in entrainment of circadian clock (20, 21). These genes, activated by light, encode transcriptor factor proteins involved in molecular mechanism of resetting the circadian clock (20). Among these genes, are *c-fos* and *nur 77*, two of the early-response genes known to be induced in the SCN by light, and *egr-3*, a zinc-finger transcription factor, whose induction by light seems to be restricted to the ventral SCN, a structure involved in entrainment (22). Light also induces *Jun-B* messenger RNA expression and *AP-1* activity in the SCN (20). Moreover, other mammalian genes involved in circadian regulation, like *mper 1* and *mper 2* have been shown to be expressed in SCN under light stimulus control (23). It has been demonstrated that light stimulus induces expression of *C-fos* gene in postnatal rat retinas (24). The earliest expression occurs between postnatal days 11 and 15 and is correlated to the genes coding for proteins involved in phototransduction, suggesting that it may play a role in the regulation of these genes in retinal cells during the light/dark cycle (24). This could in part explain the severe alteration of hormonal rhythmicity in born blinds. Further evidence that genes involved in clock regulation are reset by light has been given by studies in *Neurospora* (25). In particular, the *white collar-1 (wc-1)* and *white collar-2 (wc-2)*, both global regulators of photoresponses in *Neurospora*, encode DNA binding proteins containing PAS domains and acting as transcriptional activators, thus playing an essential role in the organization of circadian rhythmicity. Similarities between the PAS domain regions of molecules involved in light perception and circadian rhythmicity in several species suggest an evolutionary link between ancient photoreceptor protein and more recently described proteins required for circadian oscillation (25, 26).

## Role of Pineal Gland and Melatonin

The effects of environmental light on the hypothalamic–pituitary–gonadal axis are mediated by the pineal gland, through melatonin secretion (27, 28). Light stimulus from the environment reaches the retina; from here, through a RHT reaches the SCN, then the superior cervical ganglion, and finally the pineal gland, where it exerts an inhibiting effect on the pineal melatonin secretion. Instead, the darkness activates alpha1 and alpha2-adrenergic receptors in pineal gland, then it increases cyclic AMP and calcium concentration and activates arylalkylamine *N*-acetyltransferase, thus initiating the synthesis and release of melatonin, whose circadian rhythmicity is under control of an endogenous free-running pacemaker located in the SCN (29). As result of the opposite effect of light and darkness, melatonin rhythm normally peaks at night both in animals and in humans (29). Light exposure at night induces a parallel reduction in both plasma and salivary melatonin (30). A little amount of melatonin may be synthesized directly by retina: melatonin synthesis in cultured neural retinas of golden hamster exhibits a circadian rhythm entrained by light/dark cycles applied *in vitro*, whereas it shows a free-running rhythm when the culture is held on constant darkness (31). Several melatonin receptors have been found and cloned in animal and in humans. They belong to a superfamily of G-protein coupled receptors and mediate the physiological actions of melatonin with different specificity (29, 32–36). Among these, of particular importance are Mel 1a, isolated in brain, SCN, and pituitary, which is involved in circadian and reproductive processes (29, 32, 34); Mel 1b, isolated in retinas and brain, which is involved in retinas physiology regulation in some mammals (33); and Mel H9, isolated in pituitary, which is likely involved in genetically based neuroendocrine disorders (35).

Blindness affects melatonin secretion significantly. Blind patients show increased day-time melatonin levels or more complex changes in circadian rhythmicity (36–39). They exhibit a phase-advanced or a phase-delayed rhythm with respect to that of normal subjects. However, the exposure to bright light may suppress the high melatonin levels in some blind subjects with functional integrity of the RHT (40, 41). In fact, their melatonin secretion may be suppressed when their eyes are exposed to a bright light stimulus. Interestingly, these patients were less suffering for sleep alterations. The authors who studied these patients concluded that some blind people can have a functional integrity of RHT, allowing a melatonin suppression when exposed to light stimulus and consequently a sufficient sleep entrainment. Instead, blind patients with complete absence of bright input to the circadian system may represent a distinct form of blindness, associated with periodic insomnia correlated to abnormalities of melatonin rhythm, due to the persistent lack of synchronizing effect of light (40). In fact, changes in melatonin rhythmicity are more severe in patients with total blindness compared to those with only light perception (42). Interestingly, a reduced incidence of cancer has been observed in blind people (43). Even if other explanations have to be considered, the protective effect of high melatonin concentrations may not be excluded (43).

## Light, Blindness, and Hypothalamic–Pituitary–Gonadal Function

Light influences favorably gonadal function in animals and this effect seems to be mediated by reduction of pineal melatonin production, whereas a reduction of photoperiod impairs this function through an activation of melatonin secretion (27, 28, 44). Sexual activity in animals is reduced during the months of the year with short day; this reduction is prevented by pinealectomy (28, 44). Moreover, increased melatonin levels and reduction of plasma luteinizing hormone (LH), follicle-stimulating hormone (FSH), prolactin (PRL), T levels, testis weight, spermatozoa production, and sexual activity have been documented in animals rendered blind or exposed to a short photoperiod (44–48). These effects are prevented by pinealectomy (28, 45). Seasonal variations in luminosity influence melatonin secretion and some functions correlated not only in animals (28) but also in humans. Women living in Finland, a region with a strong seasonal contrast in luminosity, showed increased melatonin and reduced gonadotropin secretion during dark season, with consequent reduction of conception rates (49). Seasonal variations of plasma LH and T concentrations have been demonstrated also in patients with primary and secondary hypogonadism, but with peak of values in season different from that of normal subjects (18). A possible negative feed-back mechanism between melatonin and hormones of pituitary–gonadal axis seems to be suggested by the presence of gonadotropin and gonadal steroid receptors in human pinealocytes (50) and conversely of melatonin receptors in human hypothalamus, pituitary, and in other tissues of gonadal tract (51). Other findings, instead, suggest that there is no classic feed-back between the pineal gland and the testes (52) and that administration of exogenous melatonin does not impair pituitary–gonadal hormone secretion in men (53); on the contrary it seems to amplify pulsatile LH secretion in women (54). However, this is in contrast with that occurring in patients with chronic endogenous melatonin increase that may show alterations of menstrual cycle in case of women (28, 55) and oligospermia or azoospermia in case of men (56).

Blindness can influence gonadal function in humans. Data on the age of puberty onset and fertility in blind women are conflicting. Menarche in blind girls has been described as being advanced or delayed (57–59) and fertility in adult women as being normal or impaired (60, 61). Some blind adult patients showed a normal secretory rhythm of LH, FSH, and T in spite of impaired cortisol rhythm (62). However, in this study, the majority of patients had become blind from 14 years onward, an age in which mechanisms involved in pubertal development and gonadal function are quite completed. Instead, in a group of institutionalized blind boys, whose blindness was started in the first years of life, we found impaired basal and stimulated plasma levels of LH, FSH, PRL, and T (63). Since similar alterations had been described both in hypogonadotropic hypogonadism and in delayed puberty (64, 65), several years ago we studied the same hormonal pattern in a group of institutionalized adult blind males aged 20–29. They were divided in two subgroups: 14 with total blindness and 21 with only light perception, whose age of onset of impaired vision was reported by them as the first 5 years of life (36). Both subgroups showed increased plasma melatonin levels in comparison with a normal control group of sighted subjects, but normal LH, FSH, PRL, and T levels. However, the finding of a significant increase of FSH/LH ratio in both subgroups of blind patients versus the control group, could indicate a possible subclinical impairment of testicular function that however should be verified with studies of dynamic hormonal secretions and of seminal patterns, which the patients did not consent.

In conclusion, taking into account the data appeared in the literature and the results of our previous studies, light stimulus seems to influence favorably gonadal function both in animals and in humans, likely through inhibition of melatonin secretion. Instead, the lack or reduction of light stimulus in humans can induce:

– increased plasma melatonin concentrations;
– impairment of gonadotropins, PRL, and T secretion in prepubertal blind boys causing delayed puberty or more severe hypogonadism;
– impairment of pubertal development in young blind girls and of ovarian function and fertility in blind adult women.

These alterations seem to be more severe when the blindness occurs in the first years of life.

## Conflict of Interest Statement

The authors declare that the research was conducted in the absence of any commercial or financial relationships that could be construed as a potential conflict of interest.

## References

[1] Patton DF Mistlbergero RE Circadian adaptations to meal timing: neuroendocrine mechanisms. Front Neurosci (2013) 7:185 10.3389/fnins.2013.00185 24133410 PMC3796263

[2] Maruska KP Femald RD Social regulation of gene expression in the hypothalamic-pituitary–gonadal axis. Physiology (Bethesda) (2011) 26:412–23 10.1152/physiol.00032.2011 22170959

[3] Lewy AJ Sack RL Latham JM Melatonin and the acute suppressant effect of light may help to regulate circadian rhythms in humans. In: Arendt J Pevet P, editors. Advances in Pineal Research. London: Libbey (1991). p. 285–93

[4] Czeisler CA The effect of light on the human circadian pace-maker. Ciba Found Symp (1995) 183:254–90 7656689 10.1002/9780470514597.ch14

[5] Bellastella A Pisano G Iorio S Pasquali D Orio F Venditto T Endocrine secretions under abnormal light/dark cycles and in the blind. Horm Res (1998) 49:153–7 10.1159/000023163 9550117

[6] Welsh DK Takahashi JS Kay SA Suprachiasmatic nucleus: cell autonomy and network properties. Annu Rev Physiol (2010) 72:551–79 10.1146/annurev-physiol-021909-135919 20148688 PMC3758475

[7] Dunlap JC Molecular bases for circadian clocks. Cell (1999) 96:271–90 10.1016/S0092-8674(00)80566-8 9988221

[8] Schibler U Sassone-Corsi P A web of circadian pace-makers. Cell (2002) 111:919–22 10.1016/S0092-8674(02)01225-4 12507418

[9] Doi M Hirayama J Sassone-Corsi P Circadian regulator CLOCK is a histone acetyltransferase. Cell (2006) 125:497–508 10.1016/j.cell.2006.03.033 16678094

[10] Hyrayama J Sahar S Grimaldi B Tamaru T Takamatsu K Nakahata Y CLOCK mediated acetylation of BMAL1 controls circadian function. Nature (2007) 450:1086–90 10.1038/nature06394 18075593

[11] Yan J Wang H Liu Y Shao C Analysis of gene regulatory networks in the mammalian circadian rhythm. PLoS Comput Biol (2008) 4:e1000193 10.1371/journal.pcbi.1000193 18846204 PMC2543109

[12] Dibner C Schibler U Albrecht U The mammalian circadian timing system: organization and coordination of central and peripheral clocks. Annu Rev Physiol (2010) 72:517–49 10.1146/annurev-physiol-021909-135821 20148687

[13] Sassone-Corsi P Commentary: the year in circadian rhythms. Mol Endocrinol (2010) 24:2081–7 10.1210/me.2010-0359 20926591 PMC5417379

[14] Kennaway DJ Boden MJ Varcos TJ Circadian rhythms and fertility. Mol Cell Endocrinol (2012) 349:56–61 10.1016/j.mce.2011.08.013 21872642

[15] Wongchitrat P Felder-Schmitbuhi MP Govitrapong P Phansuvan-Pujito P Simonneaux V A noradrenergic sensitive endogenous clock is present in the rat pineal gland. Neuroendocrinology (2011) 94:75–83 10.1159/000327430 21525730

[16] Alvarez JD Hansen A Ord T Bebas P Chappell PE Gielbultowicz JM The circadian clock protein BMAL1 is necessary for fertility and proper testosterone production in mice. J Biol Rhythms (2008) 23:26–36 10.1177/0748730407311254 18258755 PMC2862364

[17] Kovanen L Saarkoski ST Aromaa A Lonnqvist J Partonen T ARNTL (BMAL1) and NPAS2 gene variants contribute to fertility and seasonality. PLoS One (2010) 5:e10007 10.1371/journal.pone.0010007 20368993 PMC2848852

[18] Bellastella G Pane E Iorio S De Bellis A Sinisi AA Seasonal variations of plasma gonadotropin, prolactin, and testosterone levels in primary and secondary hypogonadism: evidence for an independent testicular role. J Endocrinol Invest (2013) 36(5):339–42 10.3275/8620 23013937

[19] Sellix MT Clocks underneath: the role of peripheral clocks in the timing of female reproductive physiology. Front Endocrinol (Lausanne) (2013) 4:91 10.3389/fendo.2013.00091 23888155 PMC3719037

[20] Kornhauser JM Nelson DE Mayo KE Takahashi JS Regulation of Jun-B messenger RNA and AP-1 activity by light and a circadian clock. Science (1992) 255:1581–4 10.1126/science.1549784 1549784

[21] Kornhauser JM Mayo KE Takahashi JS Light, immediate-early genes and circadian rhythms. Behav Genet (1996) 26:221–40 10.1007/BF02359382 8754249

[22] Morris ME Viswanathan N Kuhlman S Davis FC Weitz CJ A screen for genes induced in the suprachiasmatic nucleus by light. Science (1998) 279:1544–7 10.1126/science.279.5356.1544 9488654

[23] Albrecht U Sun ZS Eichele G Lee CC A differential response of two putative mammalian circadian regulators, mper 1 and mper 2, to light. Cell (1997) 91:1055–64 10.1016/S0092-8674(00)80495-X 9428527

[24] Ohki K Yoshida K Harada T Takamura M Matsuda H Imaki J C-fos gene expression in postnatal rat retinas with light/dark cycle. Vision Res (1996) 36:1883–6 10.1016/0042-6989(95)00284-7 8759427

[25] Crosthwaite SK Dunlap JC Loros JJ Neurospora wc-1 and wc-2 transcription, photoresponses and the origins of circadian rhythmicity. Science (1997) 276:763–9 10.1126/science.276.5313.763 9115195

[26] Rastogi A Kumary Y Rami S Kumar V Neural correlates of migration: activation of hypothalamic clock(s) in and out of migratory state in the blackheaded bunting (*Emberiza melanocephala*). PLoS One (2013) 8(10):e70065 10.1371/journal.pone.0070065 24204554 PMC3804485

[27] Reiter RJ The pineal gland: an intermediary between the environment and the endocrine system. Psychoneuroendocrinology (1983) 8:31–40 10.1016/0306-4530(83)90039-2 6308699

[28] Reiter RJ Melatonin and human reproduction. Ann Med (1988) 30:103–8 10.3109/07853899808999391 9556096

[29] Brzezinski A Melatonin in human. N Engl J Med (1997) 16:186–95 8988899 10.1056/NEJM199701163360306

[30] McIntyre IM Noman TR Burrows GD Armstrong SM Melatonin rhythm in human plasma and saliva. J Pineal Res (1987) 4:177–82 10.1111/j.1600-079X.1987.tb00854.x 3598852

[31] Tosini G Menaker M Circadian rhythm in cultured mammalian retina. Science (1996) 272:419–21 10.1126/science.272.5260.419 8602533

[32] Weaver DR Stehle JH Stopa EG Reppert SM Melatonin receptors in human hypothalamus and pituitary: implications for circadian and reproductive responses to melatonin. J Clin Endocrinol Metab (1993) 76:295–301 10.1210/jc.76.2.295 8381796

[33] Reppert SM Melatonin receptors: molecular biology of a new family of G protein-coupled receptors. J Biol Rhythms (1997) 12:528–31 10.1177/074873049701200606 9406026

[34] Sugden D Pickering H Teh MT Garratt PJ Melatonin receptor pharmacology: toward subtype specificity. Biol Cell (1998) 89:531–7 10.1016/S0248-4900(98)80009-9 9618903

[35] Gubits AK Reppert SM Assignment of the melatonin-related receptor to human chromosome X (GPR50) and mouse chromosome X. Genomics (1999) 55:248–51 10.1006/geno.1998.5661 9933574

[36] Bellastella A Amato G Bizzarro A Carella C Criscuolo T Iorio S Light, blindness and endocrine secretions. J Endocrinol Invest (1999) 22:874–85 10710278 10.1007/BF03343663

[37] Lewy AJ Newsome DA Different types of melatonin circadian secretory rhythm in some blind subjects. J Clin Endocrinol Metab (1983) 56:1103–7 10.1210/jcem-56-6-1103 6841552

[38] Bellastella A Sinisi AA Criscuolo T De Bellis A Carella C Iorio S Melatonin and pituitary-thyroid axis status in blind adults: a possible resetting after puberty. Clin Endocrinol (1995) 43:707–11 10.1111/j.1365-2265.1995.tb00539.x 8736273

[39] Sack RL Lewy AJ Blood ML Keith LD Nakagawa H Circadian rhythm abnormalities in totally blind people: incidence and clinical significance. J Clin Endocrinol Metab (1992) 75:127–34 10.1210/jc.75.1.127 1619000

[40] Czeisler CA Shanahan TL Klerman EB Martens H Brotman DJ Emens JS Suppression of melatonin secretion in some blind patients by exposure to bright light. N Engl J Med (1995) 332:6–11 10.1056/NEJM199501053320102 7990870

[41] Hatonen T Laasko ML Heiskala H Alila-Johanson A Sainio K Santavuori P Bright light suppresses melatonin in blind patients with neuronal ceroid-lipofuscinoses. Neurology (1998) 50:1445–50 10.1212/WNL.50.5.1445 9596003

[42] Lockley SW Skene DJ Arendt J Tabandeh H Bird AC Defrance R Relationship between melatonin rhythms and visual loss in the blind. J Clin Endocrinol Metab (1997) 82:3763–70 10.1210/jc.82.11.3763 9360538

[43] Feychting M Osterlund B Ahlbom A Reduced cancer incidence among the blind. Epidemiology (1998) 9:490–4 10.1097/00001648-199809000-00004 9730026

[44] Reiter RJ Pineal melatonin: cell biology of its synthesis and of its physiological interactions. Endocr Rev (1991) 12:151–80 10.1210/edrv-12-2-151 1649044

[45] Gala RR Haisenleder DJ Pieper DR Influence of blinding, olfactory bulbectomy and pinealectomy on plasma prolactin levels in the neonatally androgenized rat. Neuroendocrinology (1983) 37:9–12 10.1159/000123509 6888662

[46] Vanecek J Illnerova H Effect of short and long photoperiod on pineal acetyltransferase rhythm and on growth of testes and brown adipose tissue in developing rats. Neuroendocrinology (1985) 41:186–91 10.1159/000124176 3840235

[47] Arendt J The pineal gland: basic physiology and clinical implications. In: De Groot LJ, editor. Endocrinology (Vol. 1), Philadelphia: WB Saunders (1995). p. 432–44

[48] Olatunji-Bello II Sofola OA Effects of continuous light and darkness exposures on the pituitary-gonadal axis and thyroid activity in male rats. African J Biomed Res (2001) 4:119–22 10.4314/ajbr.v4i3.53888

[49] Kauppila A Kivela A Pakarinen A Vakkuri O Inverse seasonal relationship between melatonin and ovarian activity in humans in a region with a strong seasonal contrast in luminosity. J Clin Endocrinol Metab (1993) 76:295–301 3667880 10.1210/jcem-65-5-823

[50] Luboshitzki R Dharan M Goldman D Hiss Y Herer P Lavie P Immunohistochemical localization of gonadotropin and gonadal steroid receptors in human pineal glands. J Clin Endocrinol Metab (1997) 82:977–81 10.1210/jc.82.3.977 9062516

[51] Shang-Mian Y Niles PL Younglai V Melatonin receptors on human granulose cell membranes. J Clin Endocrinol Metab (1995) 80:1747–9 10.1210/jc.80.5.1747 7745030

[52] Ozata M Bulur M Bingol N Behyan Z Corakci A Bolu E Daytime plasma melatonin levels in male hypogonadism. J Clin Endocrinol Metab (1996) 81:18777–81 10.1210/jc.81.5.1877 8626851

[53] Luboshitzy R Levi M Shen-Orr Z Blummenfeld Z Herer P Lavie P Long-term melatonin administration does not alter pituitary-gonadal hormone secretion in normal men. Hum Reprod (2000) 15:60–5 10.1093/humrep/15.1.60 10611189

[54] Cagnacci A Elliot JA Yen SS Amplification of pulsatile LH secretion by exogenous melatonin in women. J Clin Endocrinol Metab (1991) 73:210–2 10.1210/jcem-73-1-210 1904450

[55] Reiter RJ Pineal function in the human: implication for the reproductive physiology. J Obstet Gynecol (1986) 6(Suppl 2):77–81 10.3109/01443618609081730

[56] Karasek M Pawilikowski M Nowakowska-Jankiewicz B Kolodziej-Maciejewska H Zielieniewski J Cieslak D Circadian variations in plasma melatonin, FSH, LH and prolactin and testosterone levels in infertile men. J Pineal Res (1990) 9:149–57 10.1111/j.1600-079X.1990.tb00703.x 2126039

[57] Maege K Basinski J Quarrington B Stancer HC Blindness and menarche. Life Sci (1970) 9:7 10.1016/0024-3205(70)90003-2 5435855

[58] Thomas JB Pizzarello DJ Blindness, biologic rhythms and menarche. Obstet Gynecol (1967) 30:507 6042886

[59] Zacharias L Wurtman RJ Blindness: its relation to age of menarche. Science (1964) 144:1154 10.1126/science.144.3622.1154 14148441

[60] Elden CA Sterility of blind women. Jpn J Fertil Steril (1971) 16:48–50

[61] Lehrer S Fertility of blind women. Fertil Steril (1982) 38:751–2 7141017 10.1016/s0015-0282(16)46706-3

[62] Bodenheimer S Winter JD Faiman C Diurnal rhythms of serum gonadotropins, testosterone, estradiol and cortisol in blind men. J Clin Endocrinol Metab (1973) 36:472–5 10.1210/jcem-37-3-472 4799034

[63] Bellastella A Criscuolo T Sinisi AA Iorio S Mazzuca A Parlato F Influence of blindness on plasma luteinizing hormone, follicle-stimulating hormone, prolactin and testosterone levels in prepubertal boys. J Clin Endocrinol Metab (1987) 64:862–4 10.1210/jcem-64-4-862 3102549

[64] Spitz IM Hirsch HJ Trestian S The prolactin response to thyrotropin-releasing hormone differentiates isolated gonadotropin deficiency from delayed puberty. N Engl J Med (1983) 308:575–9 10.1056/NEJM198303103081007 6402700

[65] Moshang T Jr Marxs BS Cara JF Snyder PJ The prolactin response to thyrotropin-releasing hormone does not distinguish teenaged males with hypogonatropic hypogonadism from those with constitutional delay of growth and development. J Clin Endocrinol Metab (1985) 61:1211–3 10.1210/jcem-61-6-1211 3932452

